# Winter cold-tolerance thresholds in field-grown *Miscanthus* hybrid rhizomes

**DOI:** 10.1093/jxb/erv093

**Published:** 2015-03-18

**Authors:** Murilo de Melo Peixoto, Patrick Calvin Friesen, Rowan F. Sage

**Affiliations:** University of Toronto, Department of Ecology and Evolutionary Biology, 25 Willcocks Street, Toronto, Ontario, Canada M5S3B2

**Keywords:** Cold tolerance, electrolyte leakage, LT_50_, *Miscanthus*, perennial C_4_ grasses, rhizome.

## Abstract

The cold tolerance of winter-dormant rhizomes was evaluated in diploid, allotriploid, and allotetraploid hybrids of *Miscanthus sinensis* and *Miscanthus sacchariflorus* grown in a field setting. Two artificial freezing protocols were tested: one lowered the temperature continuously by 1°C h^–1^ to the treatment temperature and another lowered the temperature in stages of 24h each to the treatment temperature. Electrolyte leakage and rhizome sprouting assays after the cold treatment assessed plant and tissue viability. Results from the continuous-cooling trial showed that *Miscanthus* rhizomes from all genotypes tolerated temperatures as low as –6.5 °C; however, the slower, staged-cooling procedure enabled rhizomes from two diploid lines to survive temperatures as low as –14 °C. Allopolyploid genotypes showed no change in the lethal temperature threshold between the continuous and staged-cooling procedure, indicating that they have little ability to acclimate to subzero temperatures. The results demonstrated that rhizomes from diploid *Miscanthus* lines have superior cold tolerance that could be exploited to improve performance in more productive polyploid lines. With expected levels of soil insulation, low winter air temperatures should not harm rhizomes of tolerant diploid genotypes of *Miscanthus* in temperate to sub-boreal climates (up to 60°N); however, the observed winter cold in sub-boreal climates could harm rhizomes of existing polyploid varieties of *Miscanthus* and thus reduce stand performance.

## Abstract (precis)

Highlight: LT_50_ values in diploid rhizomes of *Miscanthus* indicated that they retain mechanisms that confer higher tolerance of lower temperatures than occurs in the more-productive polyploid lines.

## Introduction

*Miscanthus*×*giganteus* (*Miscanthus*) is a C_4_ perennial grass that is a leading second-generation crop for bioenergy production in temperate latitudes (Beale *et al.*, 1996; Naidu *et al.*, 2003; Heaton *et al.*, 2010). *Miscanthus* is chilling tolerant and capable of rapidly developing a large canopy, allowing it to intercept a substantial fraction of sunlight early in the growing season (Zhu *et al.*, 2008; Dohleman and Long, 2009). In temperate latitudes, the peak annual biomass production of *Miscanthus* is at least twice that of switchgrass (*Panicum virgatum*), the other leading C_4_ bioenergy crop being developed for temperate climates, and is two to three times that of promising C_3_ biofuel crops (Heaton *et al.*, 2004, 2008*a*; Somerville, 2007; Zhu *et al.*, 2008). While *Miscanthus* is suitable for mid-latitudes of mild-to-moderate winters, a major question for potential growers in the expansive landscapes of the northern USA, Canada and northern Eurasia is whether it can tolerate the more severe winters present in these locations. There also remains uncertainty as to which index best predicts suitability for a particular climate region. Because it is a new crop, there has not been time to generate sufficient data from field trials to allow safe predictions regarding a region’s suitability. Survival of most winters may provide false confidence that a perennial crop is suitable for a region, because perennial stands may be destroyed by isolated extreme cold events that are not reflected in regional climate summaries. Moreover, tolerance predictions for a given region could be misleading, as climate data may not reflect the temperatures actually experienced by overwintering tissues. In the case of perennial grasses, overwintering tissues are typically below-ground rhizomes that are insulated by snow, soil, and litter, and thus would experience a different thermal regime from that reported by climate stations. To overcome these challenges, a physiologically based assessment that identifies thermal tolerance limits of overwintering tissues could provide valuable predictive data of the cold tolerance of *Miscanthus* genotypes.

Previous studies of cold tolerance in *Miscanthus* indicate that various genotypes are tolerant of only mildly subzero temperatures. In winter-dormant rhizomes of *Miscanthus*×*giganteus* harvested in January from a field site in Germany, the lethal temperature at which 50% of the rhizomes died (LT_50_) was –3.4 °C, while the LT_50_ of the diploid *Miscanthus sinensis* plants was –6.5 °C (Clifton-Brown and Lewandowski, 2000). Heaton *et al.* (2010) noted the Illinois-grown rhizomes can survive to soil temperatures as low as –6 °C. In Elora, Ontario, Canada, the *Miscanthus*×*giganteus* genotype Illinois also could survive to –6 °C but had only 10% of survivability at –8 °C (Friesen *et al.*, 2015). These lethal temperatures are relatively warm in terms of low-temperature tolerance of cold-climate plants, where cold tolerance of dormant tissues can be substantial. For example, crowns of Canadian C_4_ grasses such as *Andropogon scoparius*, *Spartina gracilis*, and *Distichlis stricta* can survive to temperatures of –27, –29, and –35 °C, respectively (Schwarz and Reaney, 1989). Dunn *et al.* (1999) showed that rhizomes of numerous varieties of *Zoysia* spp. could sprout new shoots after 2h of incubation at –10 °C, and some varieties could sprout new shoots even after treatment of –18 °C. Hope and McElroy (1990) found an LT_50_ for switchgrass (*P. virgatum*) of –22 °C; however, there was no incubation time at the target temperature, so the finding is tentative. *Spartina pectinata* has an LT_50_ of −23 to −24 °C in southern Ontario, Canada (Friesen *et al.*, 2015). These differences in cold tolerance between *Miscanthus* and northern-latitude C_4_ grasses indicate that *Miscanthus* may be poorly prepared for winter conditions in Canada and northern Eurasia. This is somewhat surprising, since wild species of *Miscanthus* survive winter at higher elevations extending into Siberia, northern Japan, and China (Stewart *et al.*, 2009; Shumny *et al.*, 2011; Yan *et al.*, 2014; Sage *et al.*, 2015). Trial plots of *M. sinensis* also show good winter survival in the colder soils of Sweden, in contrast to hybrid *Miscanthus* lines, which have poor survival in this northern location (Lewandowski *et al.*, 2000; Farrell *et al.*, 2006). These results indicate that there may be much greater genetic potential for winter cold tolerance in members of the genus *Miscanthus*, and it is possible that new lines of hybrid *Miscanthus* have exploited this potential (Deuter, 2000; Jakob *et al.*, 2009; Lee *et al.*, 2014). In this study, we evaluated the cold-tolerance thresholds of overwintering rhizomes from seven hybrid genotypes that were selected for production in higher-latitude landscapes.

To evaluate cold tolerance of overwintering rhizomes, electrolyte leakage and sprouting of rhizomes were measured following low-temperature treatment. Two cold-treatment procedures were employed. In the first, which follows typical cold-treatment protocols, the rhizomes were continuously cooled to the treatment (nadir) temperature at the rate of 1 °C h^–1^ (Peixoto, 2015). In the second, the rhizomes were cooled gradually at the rate of 1 °C h^–1^, and allowed a 24h resting stage at 2.5–4 °C intervals. The first procedure was intended to determine the cold tolerance of material in the field at the time of harvest. The second procedure evaluated the absolute cold-tolerance limit after tissues were allowed to acclimate to their greatest degree of cold hardiness (Peixoto, 2015).

## Materials and methods

### Plant material, temperature, and weather

Rhizomes of seven hybrid genotypes from crosses of *Miscanthus sacchariflorus* and *M. sinensis* were harvested from field plantations maintained by New Energy Farms (http://newenergyfarms.com) in Leamington, Ontario (42°8’21’’N, 82°38’35’’W). The varieties are currently being considered for introduction into Canada because of their high yield potential in cool climates (Dean Tiessen, late of New Energy Farms, and Dr Bill Deen, University of Guelph, personal communication). For the continuous-cooling experiment, rhizomes sampled from *Miscanthus* Amuri genotypes M115 and M147 (diploids, 2*n*=2×), genotypes M116 (Nagara) and M161 (*Miscanthus*×*giganteus* Illinois) (allotriploids, 2*n*=3×), and M118 (allotetraploid, 2*n*=4×) were assessed (Table 1). For the staged-cooling experiment, the diploids Amuri M115 and M147 were also used; however, due to a shortage of the hybrids Nagara, Illinois, and M118, the allotriploid genotype M1 and the allotetraploid M119 were examined instead (Table 1). For a comprehensive discussion of *Miscanthus* nomenclature and ploidy levels, see Hodkinson *et al.* (2002).

**Table 1.** Miscanthus genotypes used in the cold-tolerance experiments All genotypes are hybrids of *M. sinensis*×*M. sacchariflorus* and were provided by New Energy Farms of Leamington, Ontario. Source information for each genotype is listed below.

Genotype	Ploidy^*a*^	Fertility^*b*^	Experiment
M115 (Amuri)^*c*^	2n=2x	Fertile	Continuous and staged cooling
M147 (Amuri)^*c*^	2n=2x	Fertile	Continuous and staged cooling
M116 (Nagara)^*d*^	2n=3x	Sterile	Continuous cooling
M161 (Illinois)^*e*^	2n=3x	Sterile	Continuous cooling
M1^a^	2n=3x	Sterile	Staged cooling
M118^d^	2n=4x	Sterile	Continuous cooling
M119^d^	2n=4x	Sterile	Staged cooling

^*a*^ Based on unpublished flow cytometry data by Patrick Friesen and Dr Paul Kron.

^*b*^ Rosser (2012).

^*c*^ The *M. sacchariflorus* maternal parents are from the Amuri river basin in north-east Asia. The paternal parent is *M. sinensis* of unknown origin (New Energy Farms, personal communication).

^*d*^ Bred on the Island of Crete from the same *M. sacchariflorus* maternal parent line that originated from around the Nagara River in Japan (New Energy Farms, personal communication).

^*e*^ Originally collected from the Chicago Botanical Gardens with a probable origin in southern Japan (Heaton *et al.*, 2008*a* ).

Soil temperatures were monitored at the field site in order to characterize the thermal environment of the rhizomes prior to sampling. To monitor the soil temperature, thermistors were attached to Hobo U23-003 two-channel dataloggers (Onset Hobo Data Loggers, Bourne, MA, USA, http://www.onsetcomp.com). In the winter of 2009–2010, six dataloggers were spaced at least 30 m apart with one thermistor placed 1cm deep and the other 7cm deep in the soil at the site of a rhizome mass for the genotypes in the study. These depths corresponded to where most of the rhizomes occurred. A weather station (Hobo Micro Station data logger H21-002) in the centre of the field site recorded the air temperature, solar radiation, and wind speed. During the winter of 2010–2011, eight dataloggers were spaced 20 m apart and an OWL2pe weather station (EME systems, Berkeley-CA, USA, http://www.emesystems.com) was used to measure the air temperature. In addition, meteorological data was collected from Environment Canada, Kingsville station (Environment Canada 2010, http://weather.gc.ca/), 11 km distant from the field site.

To evaluate the potential for Canadian landscapes to support *Miscanthus* cultivation, we examined the data records for soil temperature at a 5cm depth, which were compiled between 1985 and 2006 by Environment Canada 2014 (http://weather.gc.ca/) from 84 weather stations at 73 locations across Canada. The stations reported between 3 and 22 years of recorded data. The lowest soil temperature reported between 1984 and 2006 was identified and is reported with the associated air temperature and snow pack in Supplementary Table S1 at *JXB* online for all 73 locations.

All artificial freezing experiments were conducted using Thermotron programmable test chambers (two of model 8200 and one model 2800 chamber; Thermotron, Holland, MI, USA, http://www.thermotron.com) and following procedures evaluated by Peixoto (2015).

Rhizomes were sampled from 3- to 4-year-old plants growing in a sandy-loam soil near Leamington, Ontario. At each collection time, six plants of each sampled genotype were randomly chosen and harvested by digging below the rhizome cluster and shaking the dirt from the cluster. In the winter, pick axes were used to trench around the plants in order to lift them out of the frozen soil. Sampled root masses were bagged and transported to the University of Toronto for cold treatment, with each plant providing a pair of rhizomes for each treatment. One rhizome was used for the electrolyte leakage assay and the other for the regrowth assay. Before being placed in the programmable freezer, rhizome pairs were covered with moist soil in trays that were subsequently enclosed in plastic wrap. In the winter, rhizome temperature during transport to Toronto was near –1 °C. In August 2010, rhizomes were near 27 °C during transport. For the continuous-cooling rate experiment, treatments were randomly assigned to each of the three programmable freezers. Rhizomes were stored at –1 °C (or 21 °C in August 2010) until their treatment started. No rhizome was stored for longer than 5 days. For the staged-cooling rate experiment, rhizomes were immediately moved to the programmable freezers after placement in the soil trays and the experiment started. Plants were sampled during November 2009, January 2010, and August 2010 for the continuous-cooling trial, and in January 2011 and February 2011 for the staged-cooling experiment. During the artificial freezing experiments, rhizome temperature was monitored using 36-gauge copper/constantan thermocouples that were inserted 4mm deep into seven rhizomes (at least one rhizome of each genotype). Thermocouples were measured with either the Thermotron 8200 datalogger or a Veriteq Spectrum 1700 thermocouple datalogger (Veriteq Instruments Inc., Richmond, BC, Canada, http://www.vaisala.com). Rhizome temperature profiles during a freezing experiment are shown by Peixoto (2015).

### Continuous-cooling experiment

Rhizome temperature was lowered below the storage temperature (–1 °C for winter samples and 21 °C for the August 2010 samples) to the treatment (nadir) temperature at 1 °C h^–1^. They then were incubated for 16h at the treatment temperature before being warmed to 21 °C at 1 °C h^–1^. The treatment temperatures used for the November 2009 and January 2010 harvests were –1, –5, –10, –15, –20, –25, and –30 °C, and 9, 4, –5, –10, and –15 °C for the August 2010 samples. After warming, one set of rhizomes was used for the electrolyte leakage measurement and another for regrowth assays. The order of treatments and the Thermotron used for each treatment were randomly established. Seven days were required to complete all treatments within a trial.

### Staged-cooling trials

For the staged-cooling trials, all rhizomes were placed inside a Thermotron 8200 freezer at –1 °C. The temperature of the chambers was then lowered to –2.5 °C at the rate of 1 °C h^–1^ and then incubated at this temperature for 24h. The temperature was once again lowered at the same cooling rate to –5 °C. After 24h at this temperature, rhizome pairs were sampled and the samples were transferred to the Thermotron 2800 set at the treatment temperature and then thawed at 1 °C h^–1^ to 21 °C. The temperature of the Thermotron 8200 was sequentially lowered to –7, –10, –12, –14, –18, and –22 °C at 1 °C h^–1^ and the cycle (incubation at the nadir temperature followed by sampling) repeated at each of these treatment temperature (except that the –22 °C treatment was only tested in February 2011). After thawing, one rhizome of the pair was used for the electrolyte leakage measurement and the other for regrowth assays. The complete trial lasted 14 days.

### Electrolyte leakage and regrowth assays

The electrolyte assay consisted of incubating a rhizome sample in a vial containing 7ml of deionized water for 24h. After this period, an Ultrameter 4P conductivity meter (Myron L Company, Carlsbad, CA, USA) was used to determine the electrolyte conductivity of the incubated solution (electrolyte leakage of the sample, EL_sample_). Tissues damaged by cold released greater levels of electrolytes, thus raising the solution conductivity (Murray *et al.*, 1989; Peixoto, 2015). The incubation solution was then returned to the vial (containing the sample) and boiled for 1h to release all the electrolytes into the bathing solution. Once the temperature of the bathing solution returned to room temperature, the electrolyte conductivity was again measured (EL_total_). The relative conductivity is given by the formula 100×EL_sample_/EL_total_.

Regrowth assays were conducted in a greenhouse at about 26/20 °C day/night temperature, by planting the second rhizome from a sample pair in soil (40% Pro-mix, 30% loam, and 30% sand) and observing whether the rhizome grew new roots and/or shoots in the following 6 weeks. A rhizome that produced new tissue was scored as alive; one that failed to produce new tissues after 6 weeks was scored as dead.

### Experimental design and statistical analysis

In the continuous-cooling rate experiment, the experimental design consisted of replicated trials on three sampling dates (November 2009, January 2010, and August 2010). For the staged-cooling experiment, the experimental design was the same, but with just two sampling dates (January 2011 and February 2011). For each trial, rhizome clusters from six different plants per genotype were harvested randomly in the respective field plots. Each plant provided rhizomes for all temperature treatments from that trial, such that the plant was considered the unit of replication in the statistical analysis. Results of both the regrowth and electrolyte leakage assays were analysed using a logistic regression given by a generalized linear mixed-effects model on the binary distribution because survivability and relative conductivity are constrained between 0 and 1. These models were fitted using the package ‘lme4’ (Bates *et al.*, 2011) on R statistical software (R Core Team, 2013). Post-hoc means comparisons were conducted using Tukey’s test in the package ‘multcomp’ (Hothorn *et al.*, 2008). After determining the optimal model, the predicted values for rhizome survival at each temperature and relative conductivity was used to estimate the temperature (LT_50_) or relative conductivity (LEL_50_) where rhizomes had a 50% chance of mortality.

## Results

### Soil and air temperature at the field site

**Fig. 1.**
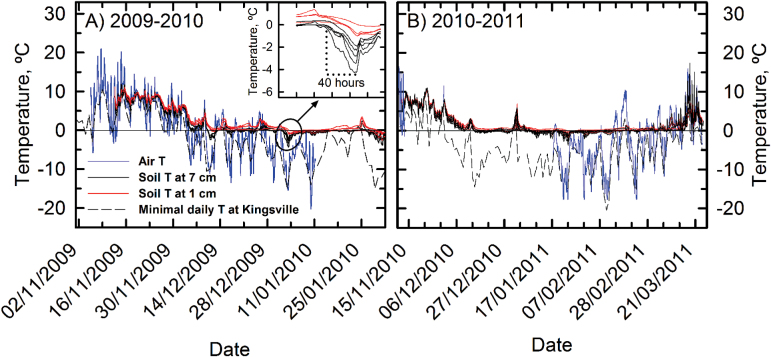
Air and soil temperature recorded for November 2009 to January 2010 (A) and November 2010 to March 2011 (B), at or near the field site in Leamington, Ontario. Thermistors recorded soil temperature at a depth of 1cm (black lines) and 7cm (red lines). The blue line shows the air temperature at the field site until 12 January 2010 (A), and November 2010 to January 2011 (B; except for 15 November 2010 to 15 January 2011: lost data). The dashed line represents the daily minimum temperature registered by Kingsville, Ontario, weather station, located 11 km from the field site (data from The Weather Office, Environment Canada, http://www.weatheroffice.gc.ca).

**Fig. 2.**
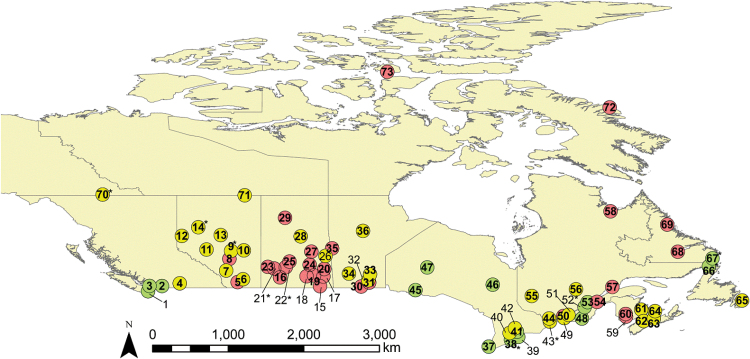
The location of 73 Environment Canada weather stations where soil temperature at a 5cm depth was recorded between 1984 and 2006. Green circles indicate weather stations where the minimal soil temperature was above –6.4 ° C, yellow circles indicate weather stations with minimal temperatures between –6.4 and –14.1 °C, and red circles indicate weather stations with minimal temperature below –14.1 °C (Environment Canada 2014; http://weather.gc.ca/). Weather stations with 5 years or less of recorded data are marked with a ‘*’. See online Supplemental Table S.1 for additional information, including the number of years recorded.

The air temperature at the Leamington, Ontario, field site decreased to –14 °C on 17 December 2009 and to –20 °C on 10 January 2010 (Fig. 1A). The soil temperature fell to the seasonal low on 3 January 2010; at 1cm below ground, soil temperature was –4 °C, while at a 7cm depth, it was –1 °C. These soil temperatures followed a warm spell in the previous week that melted the snow cover and in doing so reduced its insulation. Shortly after the observed low, new snow accumulated and the soil temperature returned to near –1 °C for the remainder of the winter. In the 2010–2011 dormant season, the coldest soil temperature recorded was –4 °C on 24 January 2011 at a 1cm depth, when the minimal air temperature fell to –15 °C and only traces of snow were present on the ground (Environment Canada 2014, http://weather.gc.ca/; Fig. 1B). The temperature database of Environment Canada 2014 (Supplementary Table S1) showed that in southern Ontario, southern British Columbia, southern Quebec, and southeast Labrador, most weather stations did not record a soil temperature below –6.5 °C at a 5cm depth between 1984 and 2006 (Fig. 2). During the same period, most stations in southwest Quebec, Nova Scotia, Alberta, the southern Yukon, and southern Northwest Territories recorded a minimum soil temperature at a 5cm depth of between –6.5 and –14 °C. Weather stations in Saskatchewan, southern Manitoba, New Brunswick, northern and eastern Quebec, western Labrador, and Nunavut recorded soil temperatures below –14 °C at a 5cm depth.

### Continuous-cooling experiment

**Fig. 3.**
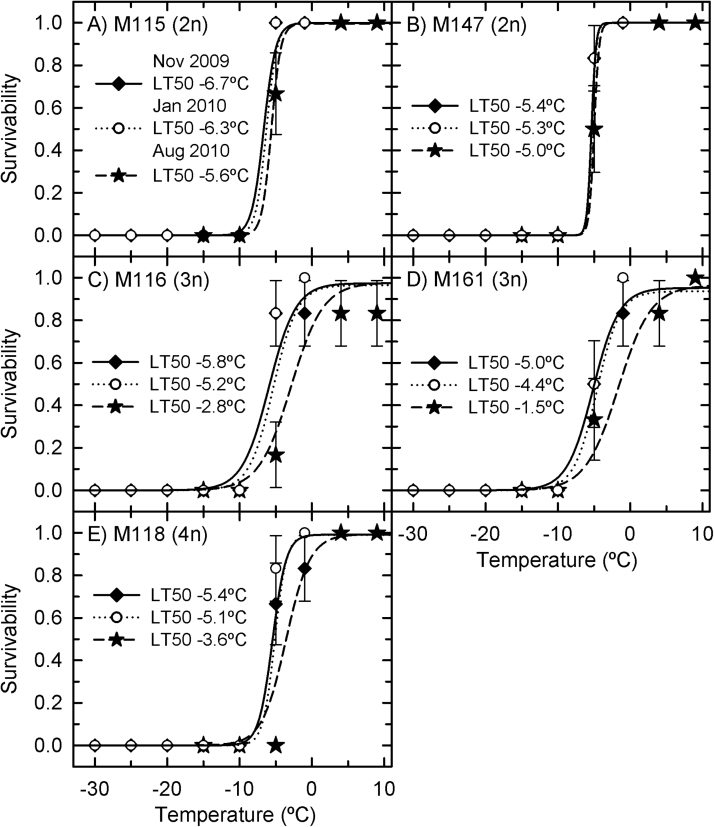
Survivability of *Miscanthus* rhizomes as a function of temperature in the continuous-cooling experiment. (A) M115; (B) M147; (C) M116; (D) M161; (E) M118. Symbols are observed values [mean±standard error (SE), *n*=6] and lines are predicted responses using a logistic regression. Filled diamonds, November 2009; open circles, January 2010; filled stars, August 2010. LT_50_ is the temperature at which a rhizome population is predicted to exhibit 50% mortality. Treatment temperature significantly affected the survivability of rhizomes (*P*<0.001), while differences between the *Miscanthus* varieties were not significant (*P*=0.53). The interaction between genotypes and temperature on survivability was significant (*P*=0.001), as was the interaction between genotype and sampling date (*P*=0.0014). Differences in sampling date were not significant (*P*=0.08).

In the continuous-cooling study, the survivability of the rhizomes from five genotypes was similar when sampled in November 2009 and January 2010 (Fig. 3). LT_50_ values from the five genotypes ranged between –4.4 and –6.7 °C. Variety Illinois (the triploid used in Illinois trials by Heaton *et al.* 2008*a* and Dohleman & Long 2009) was the least cold tolerant, with 50% rhizome mortality at –5 °, and an estimated LT_50_ of –4.4 °C in January 2010. The diploid variety M115 was the most cold tolerant, with no mortality at –5 °C for the November 2009 and January 2010 samplings and corresponding LT_50_ estimates of –6.3 and –6.7 °C. Rhizomes sampled in the summer (August 2010) were as cold tolerant as winter-sampled rhizomes in the M115 and M147 lines but were less cold tolerant by approximately 2–3 °C in the triploid (Nagara and Illinois) and tetraploid (M118) lines (Table 1). For example, for the Illinois genotype, the estimated LT_50_ in the rhizomes for the August 2010 sampling rose to –1.5 °C, approximately 3 °C warmer than for rhizomes sampled in the previous winter. In all cases, no *Miscanthus* rhizome resprouted after exposure to temperatures of –10 °C or lower.

**Fig. 4.**
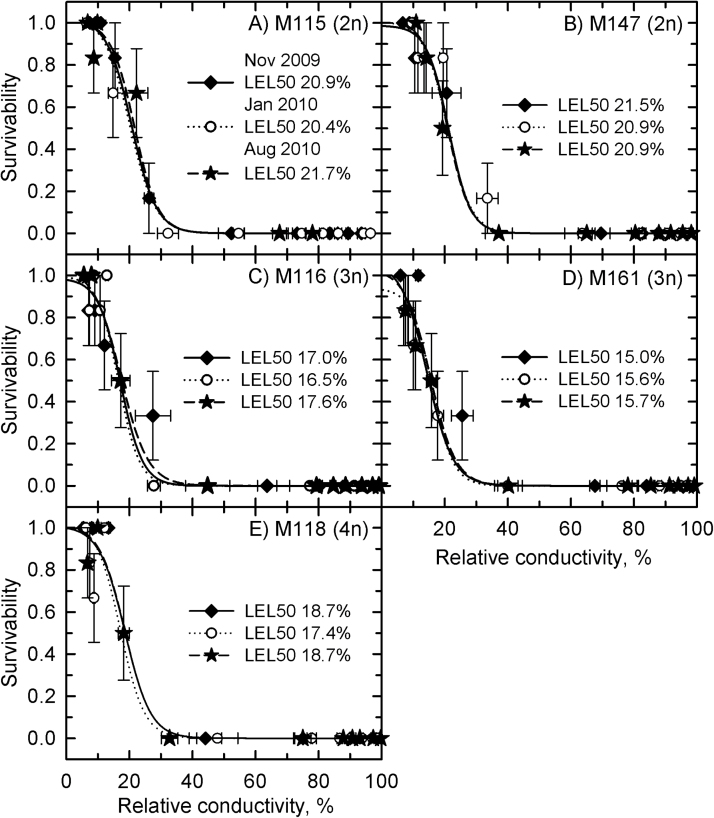
Survivability of *Miscanthus* rhizomes as a function of relative conductivity in the continuous-cooling experiment. (A) M115; (B) M147; (C) M116; (D) M161; (E) M118.Symbols are observed values (mean±SE, *n*=6), lines show predicted responses using logistic regression. Filled diamonds, November 2009; open circles, January 2010; filled stars, August 2010. LEL_50_ is the relative conductivity at which a rhizome population is predicted to exhibit 50% mortality. Collection time (*P*=0.052) and genotype (*P*=0.051) differences were not significant.

**Fig. 5.**
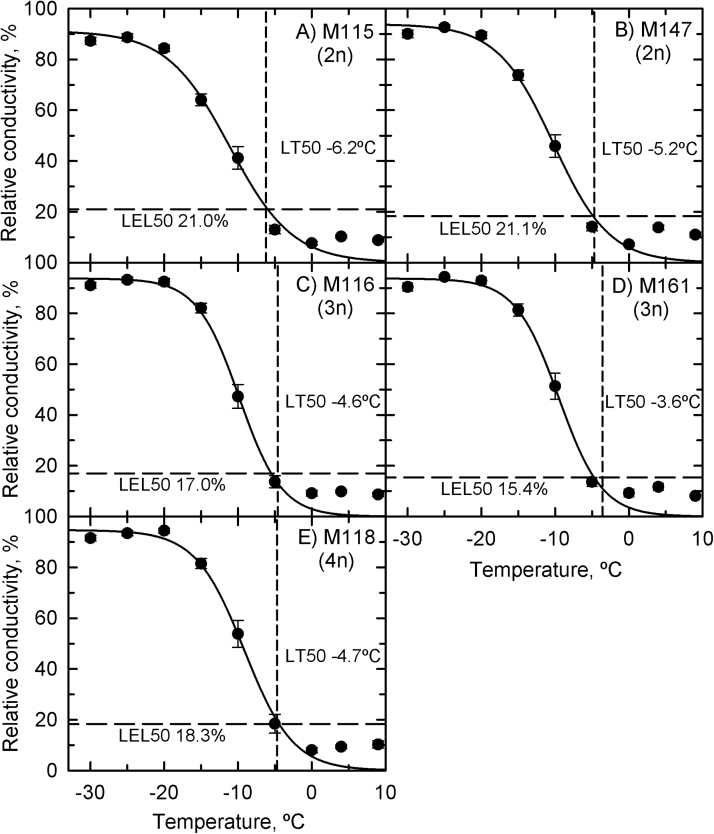
Relative conductivity as a function of the lowest temperature at which *Miscanthus* rhizomes were tested in the continuous-cooling rate experiment. (A) M115; (B) M147; (C) M116; (D) M161; (E) M118. Symbols represent mean±SE (*n*=18) of the pooled data from all collection times because there was no effect of time (*P*=0.13) or genotype (*P*=0.52). The vertical dashed line is the predicted LT_50_, and horizontal dashed line is the predicted LEL_50_. The solid line is a logistic regression.

The LEL_50_ was equivalent for all genotypes at all collection times and varied between 15 and 22% (Fig. 4). In treatments at –5 °C, the rhizomes of all genotypes exhibited lethal relative conductivity near the predicted LT_50_ values (Fig. 5). Treatment at –10 °C significantly increased the relative conductivity to above 40% in all rhizomes, none of which was viable after the treatment.

### Staged-cooling experiment

**Fig. 6.**
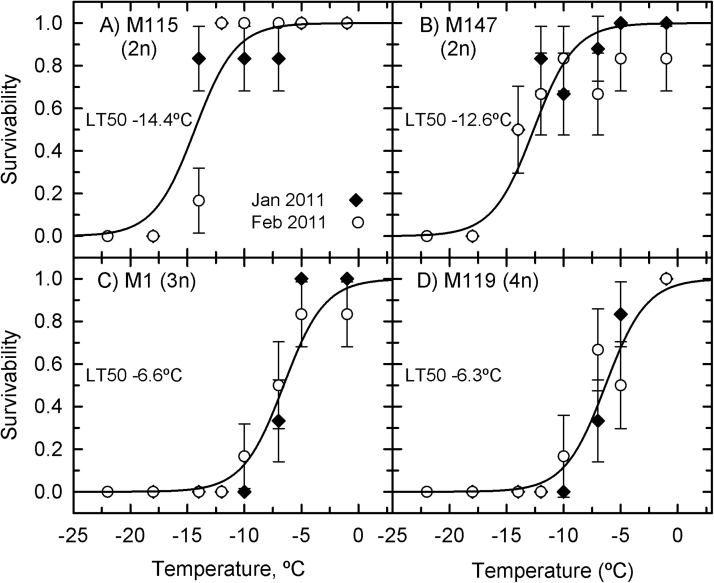
Survivability of *Miscanthus* rhizomes as a function of temperature in the staged-cooling rate experiment. (A) M115; (B) M147; (C) M1; (D) M119. Symbols represent means±SE (*n*=6) and lines represent the predicted response using a logistic regression fitted to the data. Filled diamonds, January 2011; open circles, February 2011. One regression line was drawn for both collection times because there was no difference between them (*P*=0.479). The difference in survivability between the *Miscanthus* genotypes was significant (*P*<0.001) and a post-hoc Tukey test differentiated the diploids from M1 and M119 (*P*_z_<0.001). Differences between diploids (*P*_z_=0.219) or between M1 and M119 (*P*_z_=0.993) were not significant.

When rhizomes were frozen gradually in a staged manner, rhizome survivability did not differ between the January 2011 and February 2011 collections but did differ among genotypes (Fig. 6). M115 and M147 had lower LT_50_ values (–14.4 and –12.6 °C, respectively), with no rhizome surviving below –15 °C (Fig. 6A, B). The triploid (M1) and tetraploid (M119) genotypes had similar survival patterns to the triploid and tetraploid genotypes tested in the continuous-cooling experiment, and were considerably less cold tolerant than the diploids. Their LT_50_ values were –6.6 and –6.3 °C, respectively (Fig. 6C, D). At –10 °C, no rhizome of the M1 and M119 genotypes survived in January 2011, and just one out of six rhizomes survived in the February 2011 sampling. Below –10 °C, no rhizome from M1 and M119 survived.

**Fig. 7.**
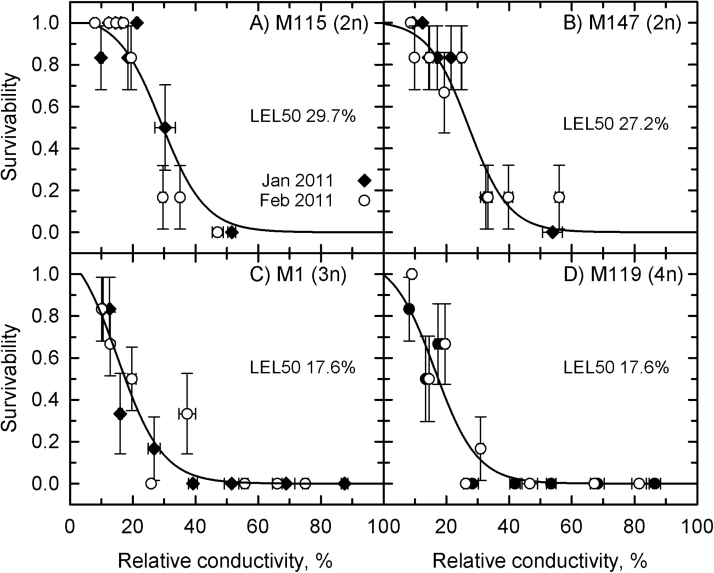
The survivability of rhizomes as a function of relative conductivity in the staged-cooling experiment. (A) M115; (B) M147; (C) M1; (D) M119. Symbols represent the observed mean±SE (*n*=6), and lines represent the predicted values fitted using a logistic regression. Differences in collection time were not significant (*P*=0.99), while genotypes showed different LEL_50_ values (*P*<0.001). The post-hoc Tukey test indicated that the diploid genotypes shared a common LEL_50_ (*P*_z_ *=0*.82), as did M1 and M119 (*P*_z_=0.99). However the LEL_50_ for M115 and M147 were different from that of M1 and M119 (*P*_z_<0.001).

**Fig. 8.**
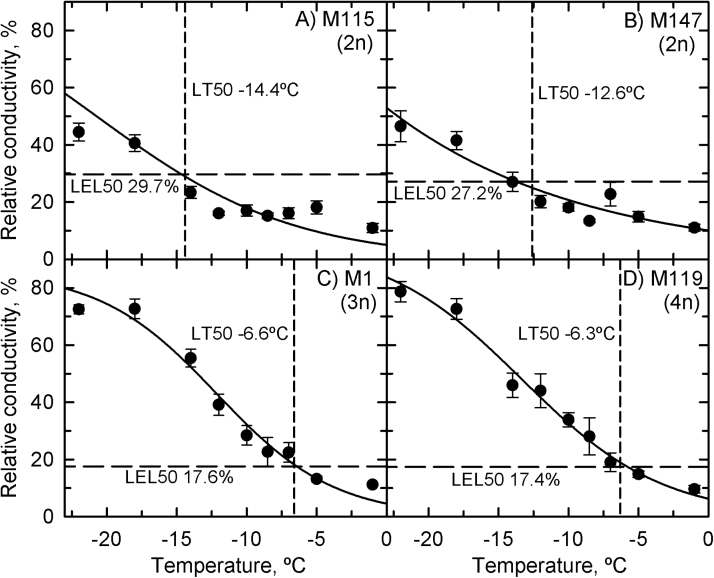
Relative conductivity as a function of the treatment temperature in Miscanthus rhizomes from the staged-cooling rate experiment. (A) M115; (B) M147; (C) M1; (D) M119. Symbols represent means±SE (*n*=12) of the pooled data from all collection times because there was no difference between collection times (*P*>0.05). Differences between genotypes were significant (*P*=0.001). The vertical dashed line is the LT_50_, and the horizontal dashed line is the LEL_50_. The solid line is a logistic regression.

The diploid genotypes also exhibited higher LEL_50_ values (30% for M115 and 27% for M147; Fig. 7A, B) in comparison with the continuous-cooling rate experiment, and with M1 and M119 (18 and 17%, respectively; Fig. 7C, D). For the staged-cooling experiment, the LEL_50_ of the diploid genotypes was reached at lower temperatures than at the continuous-cooling rate, and complete leakage was not reached even after treatment at –22 °C (Fig. 8A, B). For the allopolyploids, there was little difference in the relationship between temperature treatment and relative conductivity, and at –22 °C the rhizomes had nearly complete leakage (Fig. 8C, D).

## Discussion

Prior research has shown that *Miscanthus* rhizomes can survive down to –3.4 °C (allopolyploids) to –6.5 °C (diploids) in winter-hardened conditions (Clifton-Brown and Lewandowski, 2000). These numbers are similar to those observed when *Miscanthus* rhizomes were continuously cooled in winter at 1 °C h^–1^ (–4.4 to –6.7 °C). However, when the temperature was lowered in a more gradual, staged manner, the rhizomes of diploid hybrids of *Miscanthus* acclimated such that the lethal temperature was not observed until nearly –14 °C. Allopolyploid hybrids did not show such a shift, as they were still killed by exposure near –6.5 °C. Staged-cooled rhizomes of diploid *Miscanthus* resprouted even though they had higher electrolyte leakage than allopolyploids and continuous-cooled rhizomes, indicating greater tolerance of moderate tissue injury. These results showed that rhizomes from diploid *Miscanthus* lines have a substantial ability to acclimate to slowly decreasing winter temperatures, in contrast to rhizomes of the more-productive polyploid hybrids. Ploidy thus appears to affect potential cold hardiness, with the polyploids losing some of the genetic potential for cold tolerance that is present in the diploid lines. If widespread in the polyploid lines, this lack of tolerance to conditions colder than –10 °C could represent a significant limitation on attempts to cultivate *Miscanthus* at latitudes above the warm temperate zone. The greater cold hardiness evident in the diploid rhizomes, however, demonstrated that there is genetic potential for survival below –10 °C in the *Miscanthus* gene pool. This potential could be exploited through targeted breeding efforts or molecular transformation that introduces cold-tolerance genes into highly productive genotypes.

Cold acclimation begins before the end of the growing season, when photosynthetic rates are still high, producing sugars that will be important for subzero temperature tolerance (Gusta and Wisniewski, 2013). In *Miscanthus*, nitrogen mobilization to the rhizomes begins in August and might be an indication of the beginning of cold acclimation (Dohleman *et al.*, 2009, 2012). This first stage of acclimation is marked by the accumulation of sugars, cryoprotective compounds, anti-freeze proteins, and amino and organic acids, and modification in the membrane lipid composition (Steponkus, 1984; Thomashow, 1999). Sugar accumulation enhances cold tolerance in *Miscanthus* (Purdy *et al.*, 2013) as well as in other species including *Arabidopsis* (Kaplan and Guy, 2004, 2005; Yano *et al.*, 2005), maize (Hodges *et al.*, 1997), sunflower (Paul *et al.*, 1991), and sugarcane (Du and Nose, 2002). At subzero temperatures, sugars will modulate tissue osmolarity, thus helping to maintain the membrane stability (Jonak *et al.*, 1996; Chen *et al.*, 2014). In the continuous-cooling rate study of August 2010, the diploids had similar LT_50_ values to those measured in winter, while the rhizomes from triploid and tetraploid lines exhibited LT_50_ values that were significantly warmer than their corresponding winter values. This indicates that the diploid lines exist in a pre-hardened state during the summer, while the allopolyploids can relax their cold hardiness, which may contribute to their greater growth potential. By contrast, the results indicated that the diploid lines of *Miscanthus* retained a subzero acclimation capacity that was lacking in the allopolyploids. At subzero temperatures, a second stage of cold acclimation occurs, which is marked by cellular adjustments to deal with the effects of freezing temperatures (Weiser, 1970; Livingston III *et al.*, 2007). Such cellular adjustments prevent ice crystal formation in the protoplast, which could cause injury to the cell membrane (Levitt, 1980; Verslues *et al.*, 2006). Subzero cold acclimation was evident in the diploid *Miscanthus* genotypes because of the substantial improvement in their cold tolerance during the staged-cooling-rate experiment compared with the continuous-cooling-rate experiment. The allopolyploid genotypes did not show subzero acclimation capacity, since their LT_50_ was similar in both experiments. Identifying the physiological mechanisms enabling subzero acclimation in the diploids should identify ways to improve cold tolerance in the most productive allopolyploid *Miscanthus* genotypes.

*Miscanthus* rhizomes occur between 2 and 10cm deep in the soil, and could therefore be protected from intense winter cold by soil and snow insulation. To fully understand winter cold tolerance, it is also important to consider the degree to which cold can penetrate to rhizome depth. At the Leamington field site, the soil temperature between 2009 and 2011 never fell below –4 °C at a 1cm depth; this –4 °C extreme corresponded to a day when the mean air temperature was –15 °C and snow cover was minimal. Rosser (2012) analysed the survival rate of *Miscanthus* in the second year after establishment (in 2009) in Ontario sites at Leamington, Elora, and Kemptville. At Leamington and Elora, diploids M115 and M147, triploids Nagara and Illinois, and tetraploid M118 had winter survival above 99%. In Elora, Friesen *et al.* (2015) observed 20% rhizome mortality in the first year of establishment of the *Miscanthus*×*giganteus* genotype Illinois in an colder-than-average winter where soil temperature at a 2cm depth reached to –6 °C. This rhizome loss was concentrated in the upper region of the rhizome mass, leading Friesen *et al.* (2015) to suggest that the Elora field site, just north of Guelph, Ontario, is near the limit of where *M.*×*giganteus* can be safely grown. Consistently, in Kemptville, which is in a slightly colder climate zone than Elora, second-year stands of the diploids M115 and M147 had a survivability of 49 and 88%, respectively, while the allotriploid Nagara had 40% survivability, Illinois had 2.8%, and the allotetraploid M118 had no survivability in 2009 (Rosser, 2012). On a larger scale, the Environment Canada database of soil temperatures indicated that minimum soil temperatures at a 5cm depth did not reach lethal temperatures for rhizomes of *Miscanthus* allopolyploids in southern British Columbia, southern Ontario, and a few sites in southern Quebec and southeastern Labrador (Fig. 2). If soil cooling allows for deep cold acclimation, diploids can also be grown up to the southern Yukon and Northwest Territories, as well as in most of Alberta. However, in Saskatchewan, New Brunswick, north and eastern Quebec, and west Labrador, temperature at a 5cm depth would exceed the lethal thresholds for all the *Miscanthus* lines tested, indicating that these lines could not be safely cultivated there.

The geographic range for growing *Miscanthus* could be extended through increases in soil insulation, which could arise from genotypic selection, site selection, or altered management. Genotypic selection could result in rhizome masses that are either deeper or better insulated by the mass itself (Withers, 2014). The greater tendency to lose first-year stands of *Miscanthus* (Jones and Walsh, 2013; Arundale *et al.*, 2014*a*) may reflect less insulation present in young rhizome masses, which may be smaller, shallower, or less compact than in older plants. Site selection would result in the placement of plantations in soils with greater inherent insulation or snow cover. Soil temperature at a given depth depends on air temperature, incident solar radiation, soil cover (snow, vegetation, organic matter), soil moisture content, and type of soil (Paul *et al.*, 2004). Soil structure, density, and consistency can affect soil temperature as well. Clay/silt and moist soil have higher thermal conductivity than sandy and dry soil (AgriInfo.in., 2011). Within a given climate zone, the potential threat to Miscanthus stands can vary greatly depending on site conditions. For example, although the distance between Forêt Montmorency (site 53) and La Pocatière (site 54; Fig. 2) is relatively small (around 85 km), minimal soil temperature registered is quite large: –17.5 °C in La Pocatière and –1.5 °C in Forêt Montmorency. Differences in the snow cover (16cm vs 39cm) and mean air temperature (-23.5 °C vs –15 °C, respectively) in the 3 days prior to the measurement of the minimum soil temperature contributed to these observed differences. Management practices that moderate soil temperatures in cold winters include maintaining soil moisture, ensuring good drainage, mulching, increasing organic matter content and using fences to trap snow (AgriInfo.in, 2011). However, in regions where soil temperatures can periodically fall to lethal levels regardless of insulation properties, genetic improvement to increase tissue cold tolerance will be needed if *Miscanthus* is to contribute to the bioenergy mix in northern locations.

## Conclusion

*Miscanthus* has been identified as a potentially valuable biofuel feedstock for higher latitudes because it can maintain high productivity even at cool temperatures (Beale *et al.*, 1996; Heaton *et al.*, 2008*b*; Zhu *et al.*, 2008). The present study shows that, if subzero acclimated, diploid *Miscanthus* rhizomes can survive temperatures as low as –14 °C, which represents the coldest tolerance limit reported for any *Miscanthus* genotype. However, higher biomass yield is present in the allotriploid hybrids, which cannot survive exposure below –7 to –10°C (Lewandowski *et al.*, 2003; Clifton-Brown *et al.*, 2004; Vyn *et al.*, 2012). Because soil temperature is well buffered, not only by the soil itself but also by snow cover, there is a significant potential for *Miscanthus* to be grown successfully in Canada up to 60°N latitude. However, to safely avoid plantation loss due to episodic cold at rhizome depth, it will be necessary to transfer the greater cold tolerance of the diploid lines into the more-productive allotriploids, or to improve the insulation properties of the soil. In areas where cold penetration is substantial, it may be unwise to grow *Miscanthus*, as only one harmful year per decade is sufficient to destroy the productive potential of a perennial grass plantation. In such locations, other species such as the C_4_ species *S. pectinata* or switchgrass (*P. virgatum*), might be considered (Lee *et al.*, 2014; Friesen *et al.*, 2015). Overwintering rhizomes of both *S. pectinata* and *P. virgatum* tolerate soil temperatures down to at least –20 °C (Lee *et al.*, 2014; Friesen *et al.*, 2015); however, the yield of these species is generally half or less than the peak yield of productive *M.*×*giganteus* lines (Deen *et al.*, 2011; Arundale *et al.*, 2014*a*, *b*). Assuming the yield potential of these alternatives cannot be brought up to the *M.*×*giganteus* potential, the best strategy for realizing the C_4_ productive potential in bioenergy plantations of higher latitudes may be to identify and transfer the cold-tolerance mechanisms present in the diploid lines into the more-productive allopolyploid lines.

## Supplementary data

Supplementary data are available at *JXB* online.

Supplementary Table S1. List of the weather stations surveyed and indicated on Fig. 2, with their location, the minimal soil temperature at 5cm depth, minimal air temperature, and snow cover corresponded to the minimal soil temperature.

Supplementary Data

## Abbreviations:

EL: electrolyte leakage
LEL_50_: percentage of electrolyte leakage at which the sample has a 50% of mortality
LT_50_: temperature at which the sample has a 50% chance of mortality
SE: standard error.
