# Supplementary material for: Winter cold-tolerance thresholds in field-grown Miscanthus hybrid rhizomes
Source: J Exp Bot. 2015 Mar 18;66(14):4415–25. doi: 10.1093/jxb/erv093 (PMC4493781; doi:10.1093/jxb/erv093)
Supplement: Supplementary Data [file supp_66_14_4415__index.html]

Winter cold-tolerance thresholds in field-grown Miscanthus hybrid rhizomes — Winter cold-tolerance thresholds in field-grown Miscanthus hybrid rhizomes — Supplementary Data 

# Winter cold-tolerance thresholds in field-grown *Miscanthus* hybrid rhizomes

## Supplementary Data

Data files

**Files in this Data Supplement:**

- Supplementary Data - Supplementary Data
