# Supplementary material for: Winter cold-tolerance thresholds in field-grown Miscanthus hybrid rhizomes
Source: J Exp Bot. 2015 Mar 18;66(14):4415–25. doi: 10.1093/jxb/erv093 (PMC4493781; doi:10.1093/jxb/erv093)
Supplement: Supplementary Data [file supp_erv093_jexbot138941_file001.pdf]

Table S1. Soil temperature at 5 cm depth for 71 locations across Canada. Some locations have two weather stations for a total of 85 weather stations. Locations with multiple stations have more than one station ID. “Loc” refers to the location of the station; “Station ID” and “Stn name” are the station identifications used by Environment Canada; “Prov” refers to the province the station is located; “min soil temp at 5 cm deep” is the minimal soil temperature recorded during the years indicated; “Min air temp” is the minimal air temperature on the day the minimal soil temperature was registered (“Min soil temp reg date”); “3-day mean temp” and “snow cover” are the mean temperature and mean snow cover (respectively) on the three days before the minimal soil temperature was registered. Data compiled by Environment Canada (http://weather.gc.ca/) and supplied by Ontario Climate Centre (http://climate.weather.gc.ca/). See Fig. 2 for the location of the weather stations.

Loc	Station ID	Stn Name	Prov	Latitude (degrees)	Longitude (degrees)	Min soil temp at 5cm (ºC)	Min air temp (ºC)	3-day mean temp (ºC)	Snow cover (cm)	Min soil temp reg date	Record range	# of years
1	1016940	SAANICHTON CDA	BC	48.62	-123.42	0	-3	0.833	0	1/6/1995	1984-1996	13
2	1100119	AGASSIZ RCS	BC	49.24	-121.76	-3.3	-10.1	-6.2	NA	1/7/1993	1991-1996	6
3	1108487	VANCOUVER UBC	BC	49.25	-123.25	0	-8.3	-5.2	10	1/1/1985	1984-1990	7
4	1127800 / 112G8L1	SUMMERLAND CS	BC	49.56	-119.65	-9	-15.3	-11.33	NA	1/10/1993	1984-1996	13
5	3033890	LETHBRIDGE CDA	ALTA	49.7	-112.77	-14	-31	-25.2	3	2/3/1985	1984-1990	7
6	3036681	VAUXHALL CDA	ALTA	50.05	-112.13	-11.5	-33.5	-27.6	3	2/3/1985	1984-1992	9
7	3036652	UNIVERSITY OF CALGARY	ALTA	51.08	-114.13	-12.5	-26.5	-20.7	3	2/3/1985	1984-1990	7
8	3023720	LACOMBE CDA	ALTA	52.47	-113.75	-15	-33	-23.43	2	2/5/1988	1984-1995	12
9	3012295	ELLERSLIE	ALTA	53.42	-113.55	-8.9	-35.5	-14	21	2/3/1985	1984-1986	3
10	3016761	VEGREVILLE CDA	ALTA	53.48	-112.03	-13.9	-40.5	-31.43	NA	1/8/1991	1984-1994	11
11	3062244	EDSON A	ALTA	53.58	-116.47	-7	-23.6	-25.1	8	2/20/1986	1984-1993	10
12	3070560	BEAVERLODGE CDA	ALTA	55.2	-119.4	-12.8	-31.5	-25	6	3/2/1991	1984-1996	13
13	3066001	SLAVE LAKE A	ALTA	55.3	-114.78	-13.5	-34.2	-28.35	20	12/24/1984	1984-1993	10

14	3075040	PEACE RIVER A	ALTA	56.23	-117.45	-8.5	-22.5	-17.5	4	1/9/1984	1984-1988	5
15	4012400	ESTEVAN A	SASK	49.22	-102.97	-16	-30.8	-20.85	4	1/18/1992	1984-2008	25
16	4028040 / 4028060	SWIFT CURRENT	SASK	50.27	-107.73	-24	-34.5	-29.43	8	2/3/1985	1984-2008	25
17	4010879	BROADVIEW	SASK	50.37	-102.57	-20	-39.6	-30.23	6	1/9/1991	1985-1994	10
18	4016640	REGINA CDA	SASK	50.4	-104.57	-21	-38.5	-30.6	7.33	2/4/1985	1984-1993	10
19	4013490	INDIAN HEAD PFRA	SASK	50.5	-103.68	-15.5	-22	-16.86	2	12/10/1988	1984-1995	12
20	4019080	YORKTON A	SASK	51.27	-102.47	-19	-39.2	-34.43	22	2/3/1989	1984-1991	8
21	404037Q	BAD LAKE IHD 102	SASK	51.32	-108.42	-14.5	-35	-26.5	3	12/2/1985	1984-1986	3
22	4055736	OUTLOOK PFRA	SASK	51.48	-107.05	-16.5	-33	-28.1	1	11/27/1985	1984-1986	3
23	4043900	KINDERSLEY A	SASK	51.52	-109.18	-25	-35.2	-30.03	7	2/5/1988	1987-2006	20
24	4019035	WYNYARD	SASK	51.77	-104.2	-22	-33	-27.53	1	1/5/2004	1984-2005	22
25	4057180	SASKATOON SRC	SASK	52.15	-106.6	-18.5	-37	-32.03	10	1/9/1991	1984-1997	14
26	4083321	HUDSON BAY A	SASK	52.82	-102.32	-9	-37.8	-35.33	15	12/21/1989	1984-1993	10
27	4075518	NIPAWIN A	SASK	53.33	-104	-21	-33.6	-31.43	10	2/3/1989	1984-1997	14
28	4064150	LA RONGE A	SASK	55.15	-105.27	-11	-37.6	-30.4	0.5	11/28/1985	1984-1995	12
29	4061861	CREE LAKE	SASK	57.35	-107.13	-15	-41	-29.4	12	1/19/1984	1984-1993	10
30	5021848	MORDEN CDA	MAN	49.18	-98.08	-21	-30	-24.4	0.5	1/20/1984	1984-1998	15
31	5021054	GLENLEA	MAN	49.65	-97.12	-19	-33.5	-27.65	23	1/7/1991	1984-2002	19
32	5023222 / 5023224	WINNIPEG	MAN	49.92	-97.23	-13	-35.7	-29.46	42	1/10/1989	1984-1997	14
33	5031038	GIMLI	MAN	50.63	-97.02	-12	-32.4	-26.6	6	12/26/1984	1984-1991	8

34	5043158	MCCREARY	MAN	50.71	-99.53	-12	-24	-18.83	NA	2/17/1993	1991-1997	7
35	5052060	PASQUIA PROJECT	MAN	53.72	-101.53	-17	-40.5	-32.5	13	1/9/1991	1984-2005	22
36	5062922 / 5062926	THOMPSON	MAN	55.8	-97.86	-13.5	-34	-30.27	29	1/3/1986	1984-2007	24
37	6133360	HARROW CDA	ONT	42.03	-82.9	-4.8	-17.5	-12.86	2	1/10/1988	1984-1989	6
38	6137730	SIMCOE	ONT	42.85	-80.27	-3	-22	-16.75	14.5	1/21/1985	1984-1986	3
39	6139145	VINELAND STATION	ONT	43.18	-79.4	-4	-14.6	-9	0.33	3/7/1989	1984-1989	6
40	6142285 / 614B2H4	ELORA AUTOMATIC CLIMATE STATION	ONT	43.65	-80.42	-7.8	-24	-14.75	NA	1/14/1988	1984-1995	12
41	6158740	TORONTO MET RES STN	ONT	43.8	-79.55	-4	-16.5	-11.6	4.33	2/27/1986	1984-1988	5
42	611KBE0	EGBERT CARE	ONT	44.23	-79.78	-8	-23.5	-16.75	7	2/7/1993	1988-1995	8
43	6104025	KEMPTVILLE	ONT	45	-75.63	-11	-24	-17.53	0	3/8/1989	1988-1989	2
44	6105976	OTTAWA CDA	ONT	45.38	-75.72	-9	-21.7	-15.56	3	2/18/1989	1984-1998	15
45	6020379	ATIKOKAN	ONT	48.75	-91.62	-2	-37.6	-26.43	27.33	2/13/1988	1984-1988	5
46	6073960	KAPUSKASING CDA	ONT	49.4	-82.43	-4.9	-30	-18.36	7.33	12/21/1984	1984-2000	17
47	6016525	PICKLE LAKE (AUT)	ONT	51.45	-90.22	-3	-28.1	-24.3	67	3/5/1989	1984-1990	7
48	7024280	LENNOXVILLE	QUE	45.37	-71.82	-4	-22	-14.87	5.66	3/7/1990	1984-1995	12
49	7026839	STE ANNE DE BELLEVUE	QUE	45.43	-73.93	-11.5	-25	-17.13	18	3/8/1989	1984-1992	9
50	7025250 / 7035290	MONTREAL/	QUE	45.67	-74.03	-18.5	-30.4	-26.05	11	1/17/1992	1984-1999	16
51	7014160	L'ASSOMPTION	QUE	45.81	-73.43	-12.5	-26	-19.96	8	1/27/1992	1984-1995	12
52	7016900	ST AUGUSTIN	QUE	46.73	-71.5	-5.4	-21	-17.4	38.33	1/18/1987	1984-1987	4
53	7042388	FORET MONTMORENCY	QUE	47.32	-71.15	-1.5	-30.5	-15	39	2/1/1995	1984-1997	14

54	7054095	LA POCATIERE CDA	QUE	47.35	-70.03	-17.5	-30	-23.9	16	2/7/1993	1984-1996	13
55	7098600	VAL-D'OR A	QUE	48.06	-77.79	-9	-31.1	-24.07	43.67	2/8/1995	1984-1995	12
56	7065640	NORMANDIN CDA	QUE	48.85	-72.53	-12	-35.5	-26.26	18	2/6/1985	1984-1992	9
57	7040440	BAIE-COMEAU A	QUE	49.13	-68.2	-19.5	-30.2	-28	18.33	12/29/1993	1984-1999	16
58	7113534 / 7.113E+37	KUUJJUAQ UA	QUE	58.12	-68.42	-31.5	-42.4	-38.95	33	1/25/1994	1984-1995	12
59	8102234	HOYT BLISSVILLE	NB	45.6	-66.57	-16	-21.5	-15	0	1/27/1992	1984-1999	16
60	8101600	FREDERICTON CDA	NB	45.92	-66.62	-17	-25.5	-17.26	0	2/2/1994	1984-1999	16
61	8100592 / 8100593	BUCTOUCHE CDA	NB	46.43	-64.77	-8.5	-20.5	-16.83	13.33	2/21/1990	1984-1998	15
62	8202800 / 8202810	KENTVILLE CDA	NS	45.07	-64.48	-8	-19	-8.9	4.5	3/4/1995	1984-1998	15
63	8205990	TRURO	NS	45.37	-63.27	-8	-18	11.25	0	2/9/1997	1984-1999	16
64	8300400 / 8300401	CHARLOTTETOWN CDA CS	PEI	46.25	-63.13	-7.5	-19.2	-14.9	NA	1/28/1992	1984-1998	15
65	8403600 / 8403605	ST JOHN'S WEST CDA CS	NFLD	47.52	-52.78	-8.4	-13.7	-10.3	0.5	2/9/1998	1984-1998	15
66	8504217	WEST ST MODESTE	NFLD	51.58	-56.72	-3	-16	-12.67	NA	1/8/1985	1984-1987	4
67	8503018	PORT HOPE SIMPSON	NFLD	52.53	-56.3	-2.5	-20	-13.5	12	12/8/1985	1984-1988	5
68	8501900	GOOSE A	NFLD	53.32	-60.42	-21	-28.7	-22.76	1	2/5/1996	1984-1999	16
69	8502800	NAIN A	NFLD	56.55	-61.68	-22.5	-29.5	-25.86	172	2/10/1988	1984-1991	8
70	2101200	WATSON LAKE A	YT	60.12	-128.82	-11	-24.7	-24	19	1/1/1984	1984-1988	5
71	2202208	FORT SMITH UA	NWT	60.03	-111.93	-11.4	-42.2	-34.13	18	1/11/1999	1984-2001	18
72	2400800	CLYDE A	NU	70.49	-68.52	-39.5	-46.2	42.43	35	2/12/1987	1984-2000	17
73	2403500	RESOLUTE CARS	NU	74.72	-94.97	-25.5	-42.2	-41.86	25	3/4/1984	1984-1999	16
